# Injectate spread following posterior hip pericapsular block: a preliminary single-case anatomical observation in a formalin-fixed cadaver

**DOI:** 10.3389/fpain.2026.1867189

**Published:** 2026-05-29

**Authors:** Haotian Wu, Jianzhong Li, Nan Li, Suqin Zhang, Lei Duan

**Affiliations:** 1 School of Clinical Medicine, Tsinghua University, Beijing, China; 2 Department of Anesthesiology, Beijing Tsinghua Changgung Hospital, Beijing, China; 3 Department of Anesthesiology, Norinco General Hospital, Xi'an, China; 4 Department of Critical Care Medicine, Norinco General Hospital, Xi'an, China; 5 Department of Anesthesiology, Jiaxing Maternity and Children Health Care Hospital, Affiliated Women and Children Hospital, Jiaxing University, Jiaxing, China; 6 Department of Anesthesiology, Xi'an Aerospace Hospital, Affiliated to Northwest University, Xi'an, China

**Keywords:** cadaveric study, joint capsule, pericapsular nerve group (PENG) block, posterior hip pericapsular block (PHPB), regional anesthesia

## Abstract

**Background:**

Pericapsular nerve group (PENG) block may not reliably address posterior capsular innervation, potentially contributing to residual pain after hip surgery. The posterior hip pericapsular block (PHPB) was developed as a posterior periarticular injection to complement anterior approaches, but its anatomical spread has not been well characterized.

**Methods:**

In a formalin-fixed cadaver, ultrasound-guided PHPB was performed with injectate deposited in the fascial plane between the piriformis muscle and the ischiofemoral ligament adjacent to the posterior hip capsule. A total of 15 mL injectate, consisting of 13 mL of 0.25% ropivacaine mixed with 2 mL of 1% methylene blue, was administered. Layer-by-layer anatomical dissection was subsequently performed to assess injectate distribution along the posterior hip capsule and surrounding neural structures, including the sciatic nerve.

**Results:**

Dissection demonstrated dye distribution along the posterior hip capsule and the ischiofemoral ligament, with staining of small neural structures grossly consistent with previously described posterior articular branches. No macroscopic dye spread was observed on the superficial surface of the piriformis muscle or along the exposed sciatic nerve trunk.

**Conclusion:**

This preliminary single-case cadaveric observation demonstrated posterior pericapsular dye distribution after ultrasound-guided PHPB. The findings are descriptive and specimen-specific, and do not establish anatomical reproducibility, clinical efficacy, safety, or sciatic nerve sparing. Further validation in fresh-frozen cadavers with standardized injection protocols is warranted.

## Introduction

The sensory innervation of the hip capsule is anatomically complex, involving contributions from both anterior and posterior articular branches. Girón-Arango et al. introduced the pericapsular nerve group (PENG) block in 2018 as an anterior pericapsular technique for hip analgesia (1). However, previous anatomical studies have suggested that the posterior hip capsule receives articular innervation from branches of the nerve to the quadratus femoris as well as the superior and inferior gluteal nerves (2). These anatomical observations indicate that the posterior capsular region has a distinct pattern of neural supply that may not be fully represented by anterior pericapsular approaches alone.

The posterior hip pericapsular block (PHPB) was previously described by our group as a posterior periarticular injection technique (3). However, its injectate distribution has not been characterized in a cadaveric dissection model. Therefore, the objective of the present study was to provide a descriptive anatomical observation of injectate spread following ultrasound-guided PHPB in a single formalin-fixed cadaveric specimen. This study was designed as a preliminary, descriptive anatomical observation.

## Methods

**Figure 1:**
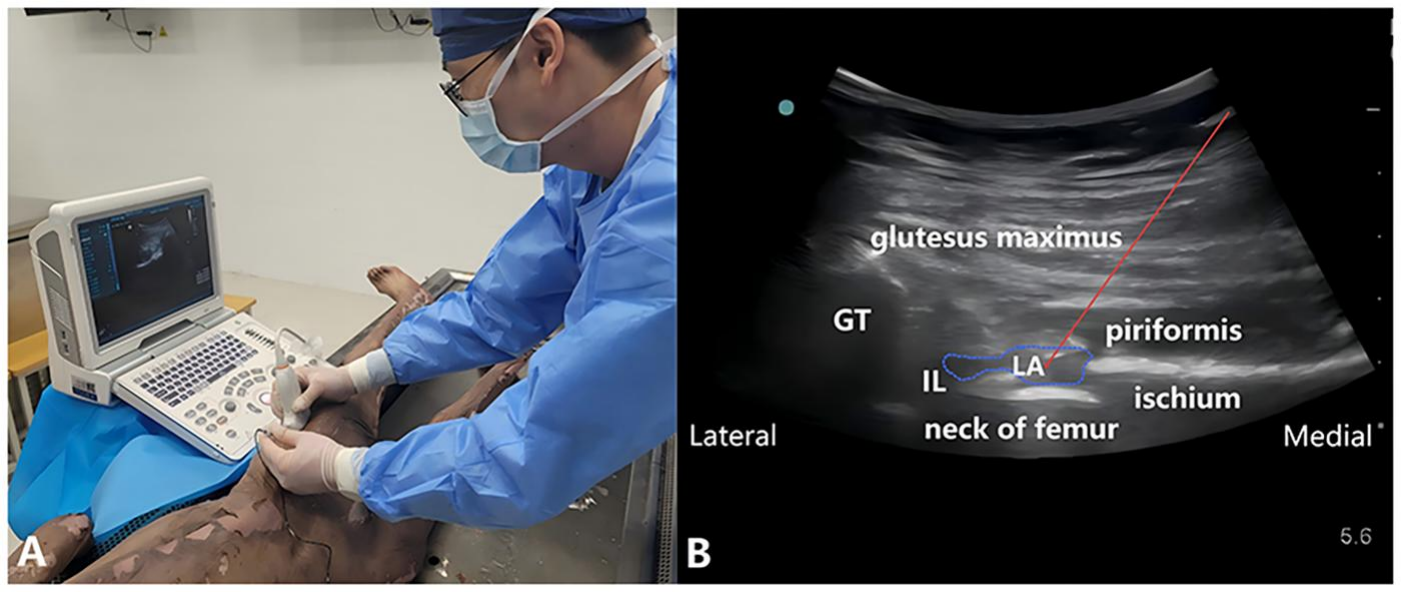
Ultrasound-guided posterior hip pericapsular block (PHPB) (right hip). **(A)** Ultrasound transducer and needle position. **(B)** Ultrasound image of the PHPB. Red arrow, needle trajectory; GT, greater trochanter; IL, ischiofemoral ligament; LA, local anesthetic.

A single formalin-fixed cadaveric specimen was used in accordance with institutional regulations governing the use of donated bodies. The cadaveric specimen was obtained from a 78-year-old male donor. The available body habitus data included a height of 1.65 m, body weight of 60 kg, and body mass index (BMI) of 22.0 kg/m^2^. The specimen had been fixed in formalin for approximately 15 months before the procedure. With the cadaver placed in the prone position, a low-frequency convex transducer (2–5 MHz) was positioned at the midpoint between the posterior superior iliac spine and the greater trochanter (Figure 1A), and then moved laterally until the piriformis muscle, ischiofemoral ligament, and greater trochanter were clearly visualized. Under in-plane medial-to-lateral ultrasound guidance, a needle was advanced into the fascial plane between the piriformis muscle and the ischiofemoral ligament adjacent to the posterior hip capsule (3) (Figure 1B). An 18-gauge, 100-mm echogenic needle was used (Contiplex type D, Braun Melsungen, Germany). A total of 15 mL of injectate, comprising 13 mL of 0.25% ropivacaine mixed with 2 mL of 1% methylene blue, was administered manually over approximately 20–30 s. This volume and injection duration were selected based on routine clinical experience with conventional sacral plexus block techniques and related posterior hip regional anesthesia procedures. Dissection was performed approximately 15 min after injection. No standardized device was used to control injection pressure, and injection force was not quantitatively measured.

The cadaver was positioned prone for anatomical dissection. The posterior hip region was exposed, and layer-by-layer dissection was performed through the skin, subcutaneous tissue, gluteal fascia, and underlying muscular planes until the posterior hip capsule and the ischiofemoral ligament were fully visualized. This approach allowed direct assessment of dye spread over the posterior capsule and adjacent neural structures, including the sciatic nerve.

## Results

**Figure 2:**
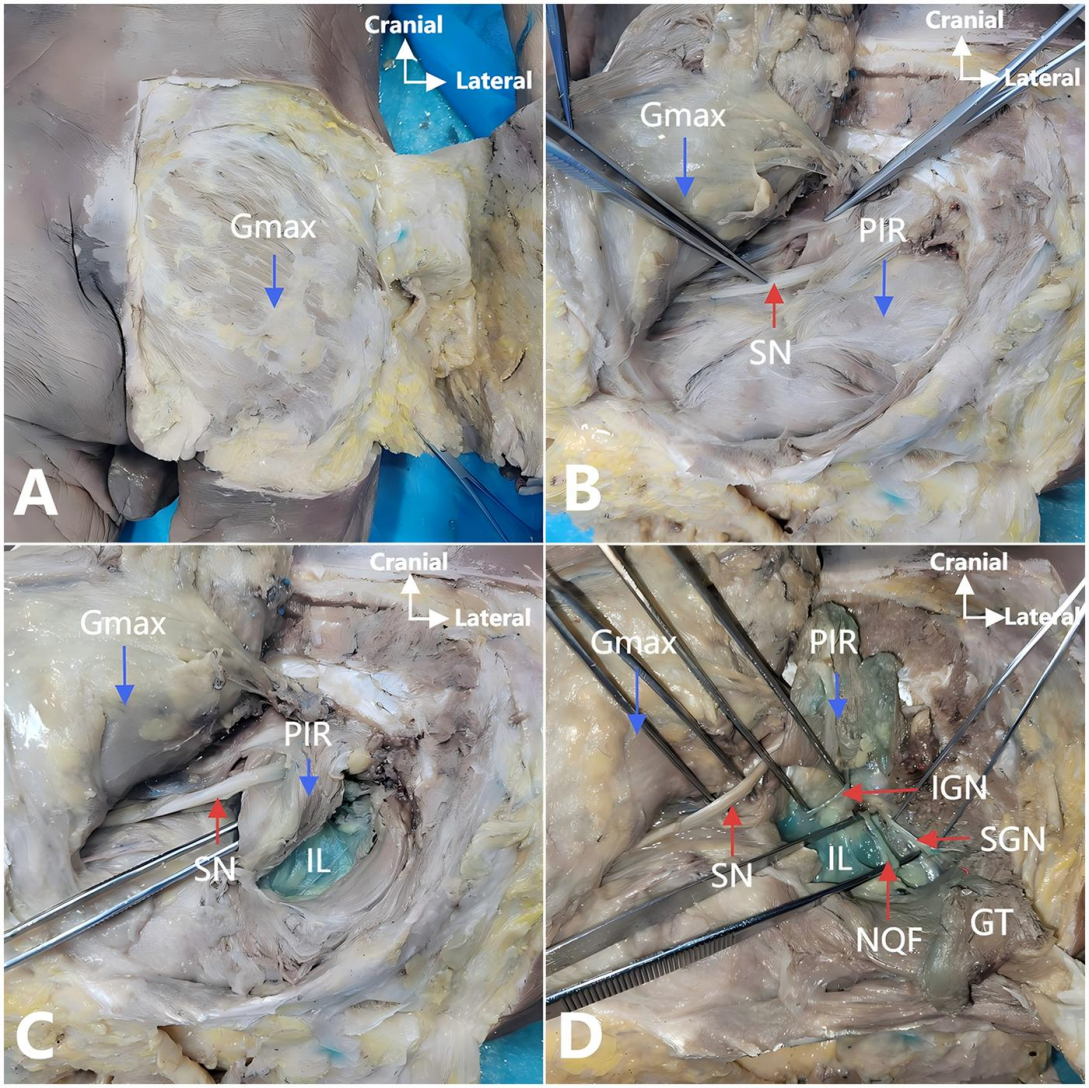
Cadaveric dissection showing methylene blue distribution after PHPB. **(A)** Superficial exposure of the gluteus maximus. **(B)** Deep surface of the gluteus maximus, showing the superficial surface of the piriformis muscle and the exposed sciatic nerve trunk without macroscopic methylene blue staining. **(C)** Deep surface of the piriformis muscle, showing methylene blue staining over the ischiofemoral ligament and adjacent posterior pericapsular region. **(D)** Small dye-stained neural structures adjacent to the posterior hip capsule, grossly consistent with posterior articular branches. Gmax, gluteus maximus; PIR, piriformis muscle; SN, sciatic nerve; IL, ischiofemoral ligament; IGN, articular branch of the inferior gluteal nerve; SGN, articular branch of the superior gluteal nerve; NQF, articular branch of the nerve to the quadratus femoris; GT, greater trochanter. Blue arrows indicate muscles; red arrows indicate neural structures.

Cadaveric dissection was performed in a layer-by-layer manner (Figure 2A). Upon reaching the deep surface of the gluteus maximus, neither the sciatic nerve nor the superficial surface of the piriformis muscle demonstrated any methylene blue staining, indicating an absence of superficial spread (Figure 2B). After transecting the lateral margin of the piriformis and reflecting the muscle superiorly, dye was observed on its deep surface, overlying the ischiofemoral ligament that contributes to the posterior hip capsule (Figure 2C). This staining pattern represents the primary distribution of the injectate, which was predominantly confined to the deep fascial plane between the piriformis muscle and the ischiofemoral ligament, with clear localization along the posterior hip capsule.

Further meticulous dissection demonstrated methylene blue staining of small neural structures adjacent to the posterior hip capsule (Figure 2D). Based on their gross anatomical location, trajectory, and caliber, these structures were considered grossly consistent with previously described posterior articular branches, including branches from the nerve to the quadratus femoris and the superior and inferior gluteal nerves. However, no histological confirmation, neural tracing, or microscopic dissection was performed; therefore, definitive neural identification was not possible. These findings should be interpreted as presumptive gross anatomical observations rather than confirmed identification of specific neural branches.

## Discussion

The posterior hip pericapsular block (PHPB) was previously described by our group as a posterior periarticular injection technique (3). The present study describes the macroscopic injectate distribution following ultrasound-guided PHPB in a single formalin-fixed cadaveric specimen. Methylene blue was observed mainly in the deep plane between the piriformis muscle and the ischiofemoral ligament adjacent to the posterior hip capsule. Additional staining was noted in small neural structures adjacent to the posterior capsule, whereas no macroscopic staining of the exposed sciatic nerve trunk was observed. These findings provide a specimen-specific anatomical description of PHPB-related dye distribution and should be interpreted within the context of the known complexity of hip capsular innervation (1, 2).

A key anatomical consideration is whether PHPB represents a distinct posterior pericapsular target rather than a nonspecific deep gluteal injection. The pericapsular nature of PHPB is based on the intended needle-tip location and injectate deposition adjacent to the posterior hip capsule, rather than merely within the broader deep gluteal compartment (3). In the PHPB approach, the needle is advanced under ultrasound guidance toward the fascial plane between the piriformis muscle and the ischiofemoral ligament, with the injectate deposited adjacent to the posterior hip capsule (3). This target is anatomically different from the parasacral interfascial plane block, which is performed in the parasacral interfascial region closer to the sacral plexus (4), and from the deep posterior gluteal compartment block, which targets the broader deep gluteal compartment (5). Therefore, PHPB may be better understood as a posterior pericapsular injection focused on the capsular side of the piriformis–ischiofemoral ligament interface, rather than as a conventional sacral plexus or deep gluteal compartment block. To clarify these anatomical and procedural differences, a comparative summary is provided in Table 1. Nevertheless, the present study cannot determine whether this anatomical target produces reproducible injectate spread across different specimens.

**Table 1:** Comparison of PHPB with PIPB and PPD block.

Technique	Main target	Procedural feature	Anatomical relationship to posterior hip capsule	Key distinction
PHPB	Plane between the piriformis muscle and ischiofemoral ligament	Can be performed with limited hip flexion	Adjacent to the posterior pericapsular region	Designed as a posterior pericapsular injection rather than a sacral plexus or deep gluteal compartment block
PIPB	Parasacral interfascial region	Posterior parasacral approach	More proximal and plexus-related	Primarily related to the parasacral/sacral plexus region rather than the posterior capsule
PPD block	Deep posterior gluteal compartment	Requires approximately 90° hip and knee flexion in the described technique	Targets a broader deep gluteal compartment	Less specifically focused on the piriformis–ischiofemoral ligament interface adjacent to the posterior capsule

PHPB, posterior hip pericapsular block; PIPB, parasacral interfascial plane block; PPD block, deep posterior gluteal compartment block.

The staining of small neural structures adjacent to the posterior capsule should be interpreted cautiously. Their location, trajectory, and caliber were grossly compatible with previously reported posterior hip articular branches, including branches from the nerve to the quadratus femoris and the superior and inferior gluteal nerves (2). However, this attribution remains anatomical and presumptive, as no histological confirmation, neural tracing, or microscopic dissection was performed. Therefore, the present study cannot definitively identify these stained structures or determine whether they would be functionally blocked *in vivo*. Similarly, the absence of macroscopic methylene blue staining on the exposed sciatic nerve trunk does not exclude microscopic dye spread or functional neural exposure, particularly in embalmed tissue, and should not be interpreted as evidence that PHPB is a sciatic-sparing or motor-sparing technique.

From an anatomical perspective, posterior pericapsular spread may be relevant to surgical contexts in which posterior capsular nociception contributes to pain, such as hip fracture surgery or total hip arthroplasty. This rationale is consistent with previous clinical interest in combining posterior pericapsular approaches with anterior pericapsular techniques for hip analgesia (3). However, this clinical projection remains speculative.

This study has several limitations. First, it was based on a single formalin-fixed cadaver, preventing assessment of inter-individual anatomical variability or reproducibility. Second, formalin fixation may alter tissue compliance, fascial permeability, and interstitial resistance; as a result, the observed dye distribution may have been constrained by the altered mechanical properties of embalmed tissue (6, 7). Previous studies suggest that fresh-frozen cadavers may better preserve tissue mechanics and more closely approximate physiological tissue properties than formalin-preserved specimens (8, 9). Third, injectate spread in fascial plane blocks is highly dependent on procedural factors, including needle-tip position, injection pressure, injection speed, tissue resistance, and injectate volume (10). In this study, although the injectate volume and injection duration were selected based on routine clinical experience with conventional sacral plexus block techniques, the injection was performed manually without objective pressure monitoring. Accordingly, the observed distribution may have been influenced by injection dynamics and should be considered potentially operator- and procedure-dependent. Fourth, neural identification was based on gross anatomical features alone and was not confirmed histologically or microscopically. These limitations prevent any generalizable conclusions regarding anatomical reproducibility, clinical efficacy, safety, or sciatic nerve sparing. Future studies using multiple fresh-frozen cadavers, standardized injection protocols, pressure monitoring, and imaging-based correlation are required before anatomical reproducibility or clinical relevance can be inferred.

## Conclusion

This preliminary single-case cadaveric observation demonstrated specimen-specific posterior pericapsular dye distribution after ultrasound-guided PHPB in a formalin-fixed specimen. Additional staining of adjacent small neural structures was observed, whereas no macroscopic staining of the exposed sciatic nerve trunk was identified. These findings should be interpreted as descriptive anatomical observations requiring further validation.

## Data Availability

The original contributions presented in the study are included in the article/Supplementary Material, further inquiries can be directed to the corresponding author.
